# Ionic Transport Aspects of Water Electrolysis in Alkaline Media

**DOI:** 10.34133/research.0788

**Published:** 2025-08-28

**Authors:** F. ElBachraoui, D. Aymé-Perrot, H. H. Girault

**Affiliations:** ^1^ Materials Science, Energy and Nanoengineering (MSN) Department, University Mohammed VI Polytechnic, Ben Guerir, Morocco.; ^2^ TotalEnergies SE, OneTech, Paris, France.; ^3^ Institute of Chemistry and Chemical Engineering, Ecole Polytechnique Fédérale de Lausanne, Lausanne, Switzerland.

## Abstract

Water electrolysis is a key industrial process for producing green hydrogen. To avoid the use of noble metals and fluorinated polymer membranes, liquid water electrolysis is often carried out in alkaline conditions. It is common to distinguish between 3 processes: alkaline electrolysis at high electrolyte concentrations (≥7 M) with porous membranes, alkaline electrolysis at high electrolyte concentrations (≥7 M) with ion-solvating membranes, and alkaline electrolysis at moderate electrolyte concentrations (<2 M) with anion-exchange membranes. Here, we consider the fundamental aspects of water and ion fluxes and of conductivity across the 3 types of membranes. We discuss ionic currents governed by ion–ion interactions and those resulting from a Grotthuss mechanism. Furthermore, in the case of porous membranes made of a polymeric fabric with compressed inorganic fillers such as zirconia, which are negatively charged in the presence of KOH, and of ion-solvating membranes such as polybenzimidazole, which also become negatively charged by deprotonation at high pH, those membranes should be a weak cation exchanger. We here address this dichotomy. All in all, we show that in all 3 cases, the membrane is an anion exchanger where hydroxide ion mobility differs from that of adjacent aqueous solution due to confinement favoring a Grotthuss-type transport and a jump mechanism.

## Introduction

Liquid water electrolysis is being considered as a key process toward the development of the hydrogen economy as part of the overall energy transition. For many, this can appear as an established and mature technology since hundreds megawatt plants of alkaline electrolysis were deployed notably during the first part of the last century, and many new large-scale projects (hundreds of megawatts and gigawatts) are announced worldwide. However, let us first remind that liquid water electrolysis encompasses many different technologies, some more mature than others, and secondly the way to operate them will evolve in comparison with historical operational ways, particularly due to the intermittent power supply of renewable energies, which brings new technical challenges. At the end of the day, there is still a lot to understand until full optimization of electrolyzer systems.

In this context, let us consider some basics within the electrolyzer cell and more particularly the case of ions. They play different roles in low-temperature electrolysis; first a thermodynamic role, as the activities of the reacting ions being either the protons given by the pH or the hydroxide anions given by the pOH define the thermodynamic redox potentials, both at the cathode and at the anode. Measuring or calculating pH or pOH values of highly concentrated solutions is a major challenge, often overlooked (see fig. S1 for KOH solutions). Then, the ions play a role as a reactant for electrode reactions where the surface concentrations determine the electrode kinetics. Finally, the ions act as current transporters in the electrolyte solutions and membranes separating the cathode and the anode.

The purpose of this paper is to discuss some fundamental questions related to the specific topic of the transport of water and ions within water electrolysis cells in alkaline conditions. We hope that this will open the pathway for new research activities to foster low-temperature (*T* < 200 °C) hydrogen production on a large scale.

**Fig. 1.**
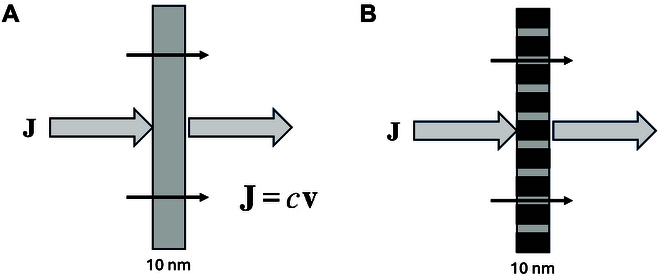
Flux through a planar surface in solution (A) and through a porous membrane (B).

First, it is important to realize that an ionic current is a flux of ions through a surface, usually consider as planar. The active area of an electrode can be much larger than its geometric area if the electrodes are nanostructured and 3-dimensional (3D). However, for the ionic current, the geometric area cannot be increased. Just to give an order of magnitude, assuming that a single ion of a monovalent salt, e.g., KOH, carries all the current, e.g., OH^−^ , a current of 10 kA·m^−2^ represents a flux of about 0.1 mol·s^−1^·m^−2^. A slab of a 1 M solution, 1 m^2^ area, and 10 nm thick, as shown in Fig. 1, contains 10^−5^ mol, and the mobile ions in the slab should therefore be replaced 10,000 times per second or every 100 μs. Accordingly, the linear speed of the mobile ions through the surface is then 0.1 mm·s^−1^ or 36 cm·h^−1^. Unlike electrodes, where the effective electrode area is often larger than the geometric one, the effective cross section of a membrane is the area of the pores through which the ions can migrate as schematized in Fig. 1. For all the membranes discussed in this article, the effective surface area is at least 10 times less than the geometric ones, meaning as the concentration remains constant that the migration velocity is at least 10 times faster, i.e., 1 mm·s^−1^ or 3.6 m·h^−1^.

A major difficulty to apprehend ionic conductivity in confined media such as a membrane is that numerical methods such as ab initio calculations provide solvation structures for a limited number of ions and molecules, and molecular dynamics simulations provide information on very short timescales, usually less than few nanoseconds. Similarly, spectroscopic techniques such as 2D infrared (IR) or small-angle x-ray scattering (SAXS) provide only short-range information. Electrochemical measurements, on the other hand, such as impedance spectroscopy, provide data on a much longer timescale, and it is rather difficult to bridge the 2 approaches to elucidate the transport mechanisms in relation to their solvation environment, in particular for the hydroxide ions.

## Porous Diaphragms and Nonselective Membranes for Alkaline Water Electrolysis

### Historical background

The oldest approach for alkaline water electrolysis, using a diaphragm, dates from the end of the 19th century. The purpose of the diaphragm was dual: to avoid the mixing of the generated gases H_2_ and O_2_ and to allow the passage of the ionic current from the anode to the cathode. By definition, a diaphragm has no ion-selective property and is just a physical barrier for the gases while being permeable to the ions and to water. Historically, inorganic diaphragms such as asbestos [chrysotile, Mg_3_Si_2_O_5_(OH)_4_] were largely used. More modern diaphragms are polymer based such as polyphenylene sulfide (PPS), polysulfones (PSU), etc., and can include an inorganic filler such as zirconia or titania particles. Alternatively, some thinner so-called ion-solvating membranes (ISMs) based on polymers such as polybenzimidazole (PBI) have recently been proposed. The field of membranes for alkali-water electrolysis and anion-exchange membrane (AEM) electrolysis has recently been thoroughly reviewed [1], and the focus of this article is dedicated to the ions transporting the current from the cathode to the anode.

Historically, the electrolyte of choice for diaphragm-based systems was KOH. The main reason was solubility, meaning a high concentration of OH^−^ in solution, as the KOH solubility in water can reach 8 M. However, the conductivity of KOH solutions reaches a maximum at about 7 M. Below this value, conductivity increases with concentrations as the number of ionic charge carriers increases, but at higher concentrations, ion–ion interactions hinder the conductivity, which decreases above 7 M also due to ion pairing effects (see fig. S2) [2]. The concentration of 7 M corresponds to a eutectic in the phase diagram water–KOH, meaning that the electrolyte would remain liquid at −60 °C, which is beneficial for electrolyzers operating in cold countries such as Norway or Switzerland. Moreover, as the viscosity of alkaline aqueous solutions typically decreases toward higher temperatures, thereby fostering ionic conductivity, alkaline electrolyzers operate in the range 80 to 90 °C.

As for the electrodes, early electrolyzers used nickel electrodes for both anode and cathode, while some modern electrolyzers used electrocatalyst-coated electrodes.

### Transport through a porous membrane

In a porous diaphragm, the pores are large enough to accommodate the surrounding KOH solutions. Ion transport is considered to occur via Brownian motion within the pores of more than 150 nm diameter. In a dilute KOH solution, the transport number of K^+^ and OH^−^ is respectively 0.27 and 0.73 (*λ*°_K+_ = 73.5 cm^2^·Ω^−1^·mol^−1^, *λ*°_OH−_ = 198.3cm^2^·Ω^−1^·mol^−1^), meaning that hydroxide ions carry about 3 quarters of the current being more mobile than hydrated potassium ions. Indeed, the transport of OH^−^ partially occurs through a Grotthuss mechanism, involving the formation and disruption of hydrogen bonds in a cluster of water molecules to effectively transport either the protons or the hydroxide ions. As the concentration increases, the evolution of the transport number could not be found in the literature, but it is likely that the hydroxide ion remains the main current carrier. As a matter of fact, it is interesting to notice that the transport number of K^+^ is 0.03 in molten KOH [3].

In electrolysis conditions, the question of the transport number of the 2 ions through the diaphragm becomes more intricated.

#### Potassium transport

Often, it is said that in the absence of advective fluxes, the flux of K^+^ across the diaphragm/membrane should be nil as it is neither produced nor consumed, considering that it is not taking part in the electrolysis reactions. The transport number should then be 0 for K^+^ and 1 for OH^−^, all the current being carried by OH^−^.

However, it should be considered that in a 7 M KOH solution, there are no “free” water molecules, and the solution is more like a “molten salt of hydrated ions” [4]. This implies that hydrated K^+^ acts also as a water carrier and proton donor for the cathodic reaction.

In this case, should the mass transfer of hydrated K^+^ through the membrane be considered as the hydrated cation becomes a reactant?

#### KOH and water diffusion

It is important to realize that the anodic reaction during water electrolysis produces water in addition to oxygen (1 mole of water to 0.5 mole of oxygen), generating a gradient of concentration of KOH across the diaphragm from the cathodic to the anodic compartment and thereby generating a diffusion flux of KOH across the membrane. This flux may also result in the creation of a diffusion potential acting against the cell voltage. A diffusion potential in solution is due to a difference of mobility between the cation and the anion. Here, the anion is faster than the cation, and all the electrostatic interionic forces ensuring the electroneutrality of the solution give rise to a potential difference and hence an electric field “pushing” the cations and “slowing down” the anions.

#### Charged pores

Another point, regarding ion current through a diaphragm, stems for the porosity of the membrane. Indeed, conductivity in porous media is highly hindered by the pore structure. Many models have been proposed in the literature for conductivity in porous media based on tortuosity, constriction factors, pore shape, electrolyte saturation [5], etc., but few deal with highly concentrated solutions. Henrique et al. [6] recently presented an interesting model describing the electrolyte transport by Kirchhoff’s laws capturing the spatial and temporal variations of charge density and electric potential. In the case of polymeric diaphragms filled with hydrophilic ZrO_2_ nano/microparticles, such as the commercial Zirfon membrane, de Groot and Vreman [7] recently measured and simulated the ohmic resistance of a zero-gap electrolysis cell and made a thorough comparison with literature data. The resistance of the Zirfon membrane was in the range of 0.1 to 0.15 Ω·cm^2^ in 30% KOH at 80 °C, corresponding to a MacMullin number of 2.8, i.e., a porosity of 50 when dealing with ideal spheres. The MacMullin number is defined as the ratio (τ^2^/ε) where τ^2^ is the tortuosity factor and ε is the porosity. The higher resistance measured in electrolysis conditions (0.23 to 0.76 Ω·cm^2^) compared to theoretical predictions has been mainly attributed to uneven current distribution and the presence of nanobubbles blocking the pores.

**Fig. 2.**
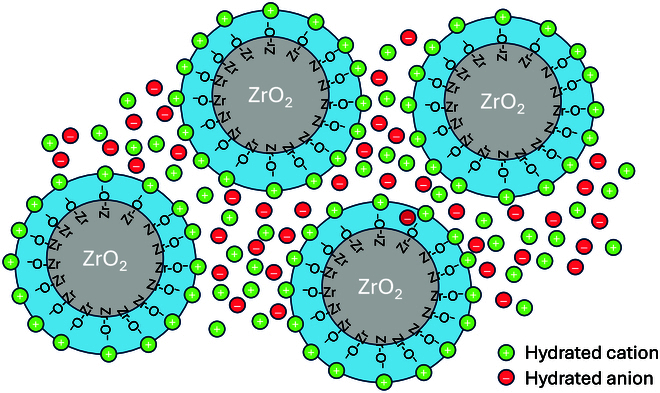
Schematic illustration of a membrane containing ZrO_2_ particles. At high pH, ZrO_2_ is negatively charged likely to be hydrated with a Helmholtz layer of rather immobile hydrated cations. In a commercial Zirfon membrane, the diameter of the particles is about 500 μm and the pore diameter is 150 nm. The electric field and hence the current lines follow the path of least resistance between the particles.

An interesting point regarding Zirfon is that the surface of ZrO_2_, being amphoteric, is negatively charged, a point that is often overlooked considering a Zirfon membrane as a neutral porous diaphragm, as for example in the work of de Groot and Vreman cited above. The ionic strength being too high for the presence of a diffuse layer, it is more likely that this negatively charged surface is compensated by the presence of a layer K^+^ cations on its surface forming a Helmholtz layer [8], as schematized in Fig. 2. In this case, can a Zirfon membrane be considered as a kind of a weak AEM rather than a weak cation exchanger?

#### Electro-osmosis

Some authors consider the presence of electro-osmosis during electrolysis. Can we have electro-osmosis in highly concentrated electrolyte solutions in the absence of “free” water molecules? Electro-osmosis is, by definition, the movement of an electrolyte solution in a capillary or porous media generated by the onset of the motion of the diffuse layer adjacent to polarized interfaces, in the presence of an electric field parallel to the surface. In dilute solutions, if we consider zirconia as negatively charged, we should have in dilute conditions electro-osmosis to the cathode generated by the influence of the electric field on the diffuse layer. Inversely, if we consider zirconia positively charged by charge inversion due to the presence of the K^+^ Helmholtz layer, do we have electro-osmosis to the anode? Inversion of electro-osmotic flow by counter ion adsorption is often used in capillary electrophoresis and other microfluidic systems to reverse the direction of the flux [9].

#### Water dragging

Often confused with electro-osmosis is the water dragging of hydrated molecules. Hydroxide anions are usually heavily hydrated (solvation number, *n* > 4) and can drag by friction many water molecules. In confined structures at highly concentrated solutions, the hydration number decreases. A surprisingly stable form has been reported [10] consisting of a rhombus hydroxide pair O_4_H_6_^2−^ [OH^−^–(H_2_O)_2_–OH^−^] instead of the tetrahydrated pyramidal structure O_5_H_9_^−^ [OH^−^–(H_2_O)_4_]. As a matter of fact, it has also been suggested that even the hydration of K^+^ in a porous diaphragm may be less strong than in the adjacent lye solution depending on the water–pore surface interaction, which can either be structure-making or structure-breaking.

#### Gas transport

The solubility of the gases cannot be neglected even if the solubility decreases sharply with the ionic strength of the solution. As discussed previously [4], the formation of nanobubbles stabilized by the presence of ions to generate an electrostatic pressure counterbalancing the Laplace pressure can also contribute to gas permeabilities. Experimentally, hydrogen permeability is higher than that of oxygen, and hydrogen crossover to the anode may generate potential risks. With the ignition threshold being 4%, hydrogen to oxygen (HTO) is generally limited to a safety level of 2% in industrial electrolyzers. In a perfect world, H_2_ permeating through the membrane should be re-oxidized at the anode to produce protons and likewise O_2_ permeating should be reduced at the cathode, just reducing the energy efficiency. However, if the oxidation of H_2_ on most anode materials is relatively fast compared to oxygen generation, the reduction of O_2_ on most cathode materials is relatively slow compared to hydrogen generation and the presence of oxygen in the produced hydrogen has also to be mitigated by post-processing using de-oxo units.

Alternatively, membrane design can be used to mitigate the gas crossover. One can cite the work of Schalenbach et al. [11] who carried out a thorough study of the influence of different physical parameters on the respective hydrogen diffusivity and electrolyte permeability. It was found that hydrogen diffusivity in pure KOH solution was more than 6 times higher than in a Zirfon PERL membrane bathing in the same solution, highlighting how the porosity of the membrane can hinder the gas diffusivity. Indeed, the tortuosity of the pores and the reduced volume fraction of the electrolyte phase are responsible for this overall decrease of hydrogen diffusivity in the membrane. They also characterized the influence of the porosity on the differential pressure-driven cross-permeation and concluded that a narrow pore membrane would be beneficial. All in all, membrane manufacturers have to find a good trade-off between reduction of gas crossover and reaching a low ionic resistance.

**Fig. 3.**
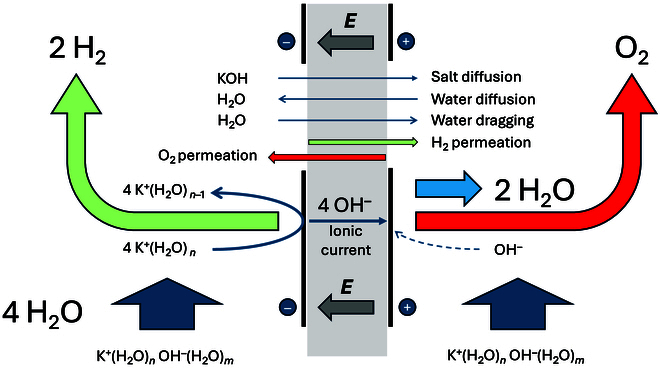
Fluxes across a diaphragm/membrane during alkaline water electrolysis.

To summarize, Fig. 3 illustrates the different fluxes through the diaphragm/membrane:

• KOH diffusion from catholyte to anolyte generated by a concentration gradient.
• Water diffusion from anolyte to catholyte generated by a concentration gradient, and perhaps in the opposite direction by water dragging by hydroxide anions or by osmotic pressure if the pressure in the 2 compartments is not balanced.
• Hydroxide anion migration from catholyte to anolyte generated by the electric field.
• Highly hydrated potassium ions by migration-diffusion from anolyte to catholyte to transport water for the cathode reaction (?)

All the fluxes are coupled to the electrode reactions, and the driving force is the potential difference applied between the 2 electrodes. Of course, a strong circulation of the lye solution on either side of the membranes may decrease the diffusion processes. All in all, it can be concluded that even if alkaline electrolysis is a mature technology, one can recognize that the question of fluxes across the membrane is complex, and some very fundamental questions regarding transmembrane transport remain open.

### Stefan–Maxwell equations for transmembrane transport

The classical methodology to analyze transport phenomena through membranes is based on irreversible thermodynamics [12]. The gist of this approach is to relate a driving force acting on a species, being charged or not, to a linear sum of frictional interactions. It was first proposed by Maxwell in the 19th century, and the resulting equations are often referred to as the Stefan–Maxwell equations. For transport processes in ionic membranes, the early work of Spiegler [13] establishes the basis of the theory. For binary electrolytes such as NaCl crossing a porous, but nonselective cellulose acetate membrane, the seminal work of Bennion and Rhee [14] provides equations not only for the flux of the different species, Na^+^, Cl^−^, and water but also for the current. It is interesting to notice that the conductivity in the membrane was less than a third that of the bulk solutions, and that the transference number of the sodium cation decreases from 0.85 at 3.5 mM NaCl concentration to about 0.3 at 75 mM. Sinha and Bennion [15] further studied the transfer of KOH through a Permion 2291 cation-exchange membrane and observed that the transport number of K^+^ decreases from 0.846 to 0.453 when the concentration of KOH was varied from 1 to 10 M, showing the loss of selectivity with increasing lye concentration. These early works clearly show that the transport numbers vary with the experimental conditions.

#### Friction coefficients

At steady state, the friction forces by the species *j* on a moving species *i* are equal to the driving force generated by a gradient of electrochemical potential

$$c_{i}\;\text{grad}\;{\tilde{\mu }}_{i}=\sum_{j}K_{\mathrm{ij}}\left(v_{j}-v_{i}\right)$$ (1)

where *c_i_* is the concentration of the species *i*, ${\tilde{\mu }}_{i}$ is the electrochemical potential, and **v** is the velocity vector. *K_ij_* is the friction coefficient of *j* on *i*. Following Onsager reciprocity law, we have *K_ij_* = *K_ji_*. We shall discuss first here the classical example of a binary electrolyte like KOH in water transporting the current through a porous membrane. In a first approximation, we shall assume electroneutrality in the solution within the porous membrane, hence equal concentration of cations and anions, defined as the concentration of KOH. The current density, **j** , through the membrane results from the opposite fluxes of the cations and anions in the membrane (see the Supplementary Materials for the definition of the terms of Eq. 1 and a full derivation of the following equations)

$$j=F\left(J_{K^{+}}-J_{{\mathrm{OH}}^{-}}\right)=\mathrm{cF}\left(v_{K^{+}}-v_{{\mathrm{OH}}^{-}}\right)$$ (2)

where *c* is the concentration of KOH, *F* is the Faraday’s constant, and **J** is the respective ionic fluxes.

Prior to electrolysis when a lye of identical composition is circulated on either side of the diaphragm, the different fluxes are nil. As soon as the electrical current is applied, the different gradients will be established and potassium transfer needs also to be considered, unless $K_{{\mathrm{wK}}^{+}}$ is very large. As shown in the Supplementary Materials, the current density can be expressed as a function of the driving forces on the ions

$$j=c^{2}F\frac{K_{{\mathrm{wK}}^{+}}\text{grad}{\tilde{\mu }}_{{\mathrm{OH}}^{-}}-K_{{\mathrm{wOH}}^{-}}\text{grad}{\tilde{\mu }}_{K^{+}}}{K_{{\mathrm{wK}}^{+}}K_{{\mathrm{wOH}}^{-}}+K_{K^{+}{\mathrm{OH}}^{-}}\left(K_{{\mathrm{wK}}^{+}}+K_{{\mathrm{wOH}}^{-}}\right)}$$ (3)

The influence of water transport from the analyte to the catholyte appears only on the friction coefficients of the solvent on the ions $K_{{\mathrm{wK}}^{+}}\;$ and $K_{{\mathrm{wOH}}^{-}}$

Considering that ion motion is only due to diffusion-migration, neglecting the osmotic and thermal effects, the driving forces for the ions are due to a gradient of concentrations and a gradient of Galvani potential *ϕ*.

$$\text{grad}{\tilde{\mu }}_{K^{+}}=\text{grad}{\text{μ}}_{K^{+}}+F\text{grad}\phi$$ (4)

$$\text{grad}{\tilde{\mu }}_{{\mathrm{OH}}^{-}}=\text{grad}{\text{μ}}_{{\mathrm{OH}}^{-}}-F\text{grad}\phi$$ (5)

The conductivity, *σ*, inside the membrane is classically defined from the current density and the electric field, **E**, acting on the membrane.

j=σE=−σgradϕ (6)

In a homogeneous porous diaphragm, the electric field can be considered in a first approximation as constant and the Galvani potential varies linearly across the membrane. From Eqs. 3 to 5, the conductivity can be calculated as a function of the different friction coefficients.

$$\frac{1}{\sigma }=\frac{1}{c^{2}F^{2}}\left(K_{K^{+}{\mathrm{OH}}^{-}}+\frac{K_{{\mathrm{wK}}^{+}}K_{{\mathrm{wOH}}^{-}}}{K_{{\mathrm{wK}}^{+}}+K_{{\mathrm{wOH}}^{-}}}\right)$$ (7)

As we have seen above, in dilute solutions $K_{K^{+}{\mathrm{OH}}^{-}}\to 0$ , and Eq. 7 reads simply

$$\sigma =c^{2}F^{2}\left(\frac{1}{K_{{\mathrm{wK}}^{+}}}+\frac{1}{K_{{\mathrm{wOH}}^{-}}}\right)={\mathrm{cF}}^{2}\left({\tilde{u}}_{K^{+}}+{\tilde{u}}_{{\mathrm{OH}}^{-}}\right)$$ (8)

We recover the classical definition of electrolyte conductivity for dilute solutions.

If we further consider that the cation–water friction is greater than that of anion–water $K_{{\mathrm{wK}}^{+}}>K_{{\mathrm{wOH}}^{-}}$, for example, considering a Grotthuss mechanism for OH^−^, Eq. 7 reduces to

$$\sigma =\frac{c^{2}F^{2}}{K_{K^{+}{\mathrm{OH}}^{-}}+K_{{\mathrm{wOH}}^{-}}}$$ (9)

Using the conductivity data of fig. S2, we can see that the conductivity increases with concentration but not in a quadratic manner, as both the ion–ion friction and the solvent–OH^−^ friction also increase. When the conductivity decreases with concentration, the dominant term in Eq. 9 is $K_{K^{+}{\mathrm{OH}}^{-}}$ simply due to the lack of free water molecules.

#### Transport numbers

The transport numbers of the ions are defined such that

$$\frac{F}{\sigma }j=t_{{\mathrm{OH}}^{-}}\text{grad}{\tilde{u}}_{{\mathrm{OH}}^{-}}-t_{K^{+}}\text{grad}{\tilde{u}}_{K^{+}}$$ (10)

They represent the fraction of the current carried by the 2 ions. From Eqs. 3 and 7, we obtain the simple expressions

$$t_{K^{+}}=\frac{K_{{\mathrm{wOH}}^{-}}}{K_{{\mathrm{wK}}^{+}}+K_{{\mathrm{wOH}}^{-}}}$$ (11)

$$t_{{\mathrm{OH}}^{–}}=\frac{K_{{\mathrm{wK}}^{+}}}{K_{{\mathrm{wK}}^{+}}+K_{{\mathrm{wOH}}^{-}}}$$ (12)

As discussed, hydroxide anions are more mobile than the potassium cations $\left(K_{{\mathrm{wOH}}^{-}}<\;<K_{{\mathrm{wK}}^{+}}\right)$and then $t_{K^{+}}=K_{{\mathrm{wOH}}^{-}}∕K_{{\mathrm{wK}}^{+}}$ is small and $t_{{\mathrm{OH}}^{-}}$ tends to unity.

The radii of K^+^ and OH^−^ are rather similar, 138 and 133 pm, respectively. However, the absolute Gibbs hydration energy of K^+^ (−335 kJ·mol^−1^) is less than that of OH^−^ (−410 kJ·mol^−1^), indicating that OH^−^ should be more hydrated with stronger ion–dipole interactions. Consequently, the ionic conductivity of OH^−^ should be smaller than that of K^+^ in a pure viscous friction model. Nevertheless, OH^−^ mobility is higher, especially during water electrolysis, and this is often explained by involving the Grotthuss mechanism even though it is less marked than for the proton. This highlights a key point regarding the water content of the membrane, as water would be a major component of hydroxide anion conductivity. In highly concentrated KOH solutions with very few free water molecules, the occurrence of a Grotthuss mechanism is questionable.

In the case of zirconia-doped porous membranes, many cations will compensate for the negative charge of the zirconia at high pH values and will therefore be quasi-static not participating in carrying the current.

In conclusion, we assert that although a porous diaphragm has no built-in ion selectivity and the negatively charged zirconia acts as a weak cation exchanger, we can conclude that hydroxide anions are the major current carriers in alkaline water electrolysis and that the transport number for K^+^ is very low.

#### KOH diffusion

If we want to consider the diffusion of KOH from the catholyte to the anolyte, we can use the definition of the chemical potential of KOH,

$$\text{grad}{\text{μ}}_{\mathrm{KOH}}=\;\text{grad}{\tilde{\mu }}_{K^{+}}+\;\text{grad}{\tilde{\mu }}_{{\mathrm{OH}}^{-}}$$ (13)

As shown in the Supplementary Materials, we obtain

$$J_{K^{+}}=-\frac{c^{2}}{K_{{\mathrm{wK}}^{+}}+K_{{\mathrm{wOH}}^{-}}}\;\text{grad}{\text{μ}}_{\mathrm{KOH}}+\frac{t_{K^{+}}}{F}j+\frac{{cJ}_{w}}{c_{w}}$$ (14)

$$J_{{\mathrm{OH}}^{-}}=-\frac{c^{2}}{K_{{\mathrm{wK}}^{+}}+K_{{\mathrm{wOH}}^{-}}}\;\text{grad}\;{\text{μ}}_{\mathrm{KOH}}-\frac{t_{{\mathrm{OH}}^{–}}}{F}j+\frac{{cJ}_{w}}{c_{w}}$$ (15)

These equations show that the flux of potassium is mainly a flux of diffusion, and a flux associated to the transport of water as $t_{K^{+}}$ is very small. The water flux can be a flux of diffusion from anolyte to catholyte or a convective flux associated to pressure difference. Conversely, the flux of hydroxide is mainly a migration flux associated to the passage of the current.

In summary, we can define the diffusion flux of KOH as

$$J_{\mathrm{KOH}}=\;J_{K^{+}}=\;J_{\mathrm{OH}-}=-\frac{c^{2}}{K_{{\mathrm{wK}}^{+}}+K_{{\mathrm{wOH}}^{-}}}\;\text{grad}{\text{μ}}_{\mathrm{KOH}}$$ (16)

It is interesting to notice in fig. S3 that at 80 °C the activity coefficient of KOH is rather constant and close to unity.

#### Water transport

It is also interesting to consider the transport of water including the diffusion of water from the analyte to the catholyte. As shown in the Supplementary Materials, we have

$$c_{w}\text{grad}{\text{μ}}_{w}=-c\text{grad}{\text{μ}}_{\mathrm{KOH}}$$ (17)

In the absence of a difference of pressure, the flux of water by diffusion is equal to the flux of KOH by diffusion from the catholyte to the anolyte. However, it should be remembered that water electrolysis produces 2 molecules of hydrogen per molecule of oxygen, and that the catholyte chamber may operate at higher pressures than the anolyte compartment. In this case, the gradient of chemical potential for water is

$$\text{grad}\mu_{w}=\mathrm{RT}\;\text{grad}\text{ l}nc_{w}+{\bar{V}}_{w}\text{grad}p$$ (18)

In the analysis above, we have neglected the friction forces between the static membrane components such as zirconia particles or the polymer support. However, the influence of zirconia is included in the friction with the adsorbed potassium ions and the friction due to the polymer is neglected considering that the hydrophobic interactions are weak.

### Membrane conductivity and resistance

#### Ionic current and membrane resistance

A classical method in membrane science is to use impedance measurements using a small AC applied voltage either in a 2- or in a 4-electrode mode, and to obtain the resistance of a membrane bathing between 2 similar electrolyte solutions by fitting the frequency dependence to a circuit representation of the measured impedance. This approach could be called resistance in quasi-equilibrium conditions. For example, for a membrane bathing in a KOH solution, the resistance will depend on the transport of both the cations and the anions. Another method consists in measuring the resistance directly under electrolysis conditions, for example, using a current interrupt approach. This is applicable to small pilot systems where the power supply can be switched on and off on a very short timescale.

In the former approach, the resistance includes contributions from both ions, whereas in the latter it will dominate by the movement of hydroxide ions. As recently discussed by de Groot and Vermeulen [16], the membrane resistance varies also with the current density, partially due to the presence of gas bubbles.

#### Conductivity in concentrated electrolytes

If the dilute electrolyte solutions can be analyzed within the framework of the Debye–Hückel and Onsager theory, there is still a lack of theories for the high concentration range beyond the Debye–Falkenhagen approach. Recently, Avni et al. [17] have proposed a stochastic density functional theory, paired with a modified Coulomb interaction that accounts for the hard-core repulsion between the ions. This approach could fit experimental data up to the 3 M range. They classically considered that the conductivity has 3 contributions [18], the limiting conductivity *σ*^o^ given by Eq. 8, an electrophoretic term, and a relaxation term. The expression for the conductivity at high concentrations expressed in terms of physical length scales is

$$\sigma \left(\lambda_{D}\right)\approx \sigma^{o\;}\left[1-\frac{r_{s}}{\lambda_{D}}e^{-a/\lambda_{D}}-\frac{1}{6}\left(1-\frac{1}{\sqrt{2}}+e^{-2a∕\lambda_{D}}-\frac{e^{-\sqrt{2}a∕\lambda_{D}}}{\sqrt{2}}\right)\frac{l_{B}}{\lambda_{D}}\right]$$ (19)

where $\lambda_{D}$ is the Debye screening length given by

$$\lambda_{D}=1∕\sqrt{8{\pi l}_{B^{c}}}$$ (20)

where *c* is the molar concentration and *l*_B_ is the Bjerrum length given by

$$l_{B}=e^{2}/4{\pi \varepsilon }_{0}\varepsilon_{r}k_{B}T$$ (21)

In Eq. 19, *r_s_* is the reduced Stokes radius dependent on the viscosity *η* and the mean electrochemical mobility

$$r_{s}=1/6\pi \eta \bar{u}$$ (22)

with

$$\bar{u}=\left({\tilde{u}}_{+}+{\tilde{u}}_{-}\right)/2$$ (23)

and *a* is the distance of closest approach of the 2 ions defined as the sum of the radii

$$a=r_{+}+r_{-}$$ (24)

The value of *a* in a simple co-sphere model $\left(a={\left(8000\pi N_{\mathrm{Av}}c/3\right)}^{-1/3}\right)$ is about 0.3 nm, which can be compared to the radii of the ions, 138 and 133 pm for K^+^ and OH^−^, respectively, leaving little space for water molecules.

This approach was expanded to deal with the Wien effect at high concentrations [19]. The first Wien effect shows that under high electric field (>10^8^ V·m^−1^), the ionic conductivity increases significantly as the moving ion breaks through its surrounding ionic cloud. The second Wien effect is the increase of conductivity of weak acids due to an increase of dissociation under high electric fields. Avni et al. [19] concluded that the Wien effect becomes more pronounced as the ion concentration increases. Again, can we consider that the hydrated potassium ion is a weak acid?

More recently, Elliott et al. [20] have discussed the so-called anomalous underscreening in concentrated solutions. In the framework of the Debye–Hückel theory, each ion is surrounded by an ionic atmosphere shell, i.e., a cloud of counter charges screening the central ion. The radius of the shell is given by the Debye length given by Eq. 20. The major consequence of this approach is that when a salt is dissolved in a solvent such as water, the ions occupy all the space being roughly equidistant between each other (cation–cation, cation–anion, anion–anion). In the 19th century, it was assumed that due to the strong coulombic interactions between cations and anions, salts in water would dissolve as ion pairs.

The gist of this Debye–Hückel model is that the central ion has little ion–ion interactions with ions outside the shell, which in effect screens it. According to the Debye–Hückel theory, the Debye length should decrease with the concentration (see Eq. 20). But as reviewed in [20], many experimental results tend to suggest that this is not true at high salt concentrations and this is often referred to as anomalous underscreening, meaning that ion–ion interactions occur over longer distances than the Debye length. Anomalous underscreening has been observed for monovalent salt in both aqueous and nonaqueous solutions, even in molten salts and ionic liquids. So, it would not be surprising to exist for highly concentrated KOH solutions. In the case of porous membranes, or even ISMs as described below, the interfaces are also known to play a role.

## Nonporous Diaphragms and Nonselective Membranes for Alkaline Water Electrolysis

### ISM resistance

Some membranes often referred to as ISMs are nonporous. These are polymer films that contain polar or ionizable groups (such as benzimidazoles or imidazoles) and that swell when immersed in KOH to achieve ionic conductivity. As diaphragms, they have no selectivity to OH^−^ transport. A key advantage of the ISMs is to alleviate some drawbacks of porous membranes, namely, pore blocking either physically when pressing the electrodes in the case of the zero-gap cell or chemically by particles floating in the lye such as iron oxide particles resulting from some corrosion processes. ISMs are often used based on low-cost arguments and good gas barrier efficiency.

**Fig. 4.**
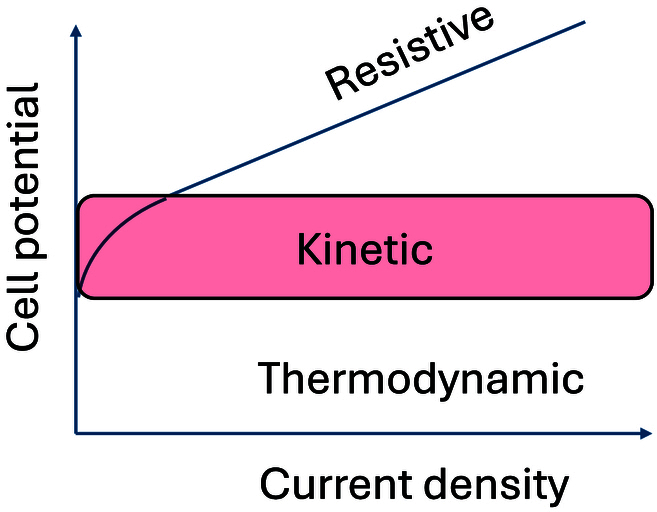
Variation of the electrolysis cell voltage with the current density. On top of the thermodynamic cell voltage, there is the kinetic overvoltage (the faster the electrode reactions, the lower the overvoltage) and the resistive regime where the overvoltage is controlled by the resistance of the solution within the membrane.

When plotting the cell voltage versus the applied current, and by comparing the linear region associated with the resistive nature of the cell, and hence of the diaphragm, it has been reported that ionic solvating membranes are less resistive than commercial membranes such as Zirfon as schematized in Fig. 4 [21].

The most obvious reason can be the membrane thickness, which can be reduced to tens of micrometers. Another more speculative reason is due to different ionic conductivity mechanisms, where the presence of perhaps more direct ion pathways may replace the tortuous classical diaphragm. Also, it could be argued that in case of hydrophobic polymeric ISMs ions are less hydrated and hence less subjected to friction forces with the water molecules. Moreover, Babcock et al. [22] showed by neutron scattering experiments on PBI-based ISMs that the polymer immediately after doping exhibits a polymer chain separation range of approximately 10 to 20 nm. The important steric hindrance in those membranes must play a key role on the resulting hydration structure.

Recently, a wide range of new approaches to membrane fabrication has been proposed. One can cite an ultrathin PPS film on reinforced PPS mesh [23] (thickness = 200 μm, resistance = 0.21 Ω·cm^2^, cell voltage = 2.33 V at 500 mA·cm^2^) or a polyvinyl alcohol (PVA)-skin layer of a Zirfon membrane [24].

A recent publication has reported a sol–gel process to produce high-performance (1,4-naphthalene)-5,50-bibenzimidazole (NPBI)-based ISM with high conductivity in low concentrations of KOH electrolyte and excellent alkaline stability (2 M KOH at 80 °C for 285 d) and high ion conductivity even in 2 M KOH solutions (190 mS·cm^−1^ at 80 °C) [25]. This NPBI membrane can further engineered to include porogen additive to enhance further the conductivity and the durability [26].

Also, novel polymer compositions have been proposed, such as poly(oxindole biphenylene)-based ISMs with highly stable oxindole/KOH complex ion pairs, able to operate more than 2,500 h at high operating electrolysis conditions such as high temperatures (≥80 °C) and voltages (2.3V) with ultralow gas permeation [27]. Progress in ISM has been rapid recently, and Dayan et al. [28] have even suggested that ISM, such as a thermally cured sulfonated para-polybenzimidazole (MS-PBI) membrane, can substitute AEMs in water electrolyzers.

Finally, a biosourced ISM based on watermelon skin is worth mentioning. Not only for water electrolysis but also for electrochemical CO_2_ reduction, where a micropore and continuous hydrogen-bonding network of cellulose fiber and pectin enable a high ion conductivity of 282.3 mS·cm^−1^ (room temperature, saturated with 1 M KOH) [29].

### Solvation in ISM

If a membrane is so-called ion solvating, it can be argued that the polymer is a solvent for the salt KOH. In this case, there must be a distribution potential difference at the aqueous electrolyte | organic polymer interface. This distribution potential is given as the half-sum of the standard ion transfer potential of K^+^ and OH^−^ [30].

$$\phi^{W}-\phi^{P}=\;\Delta_{P}^{W}\phi =\frac{\Delta_{P}^{W}\phi_{K^{+}}^{o}+\Delta_{P}^{W}\phi_{{\mathrm{OH}}^{-}}^{o}}{2}$$ (25)

where $\Delta_{P}^{W}\phi_{K^{+}}^{o}$ is the standard potassium ion transfer potential defined from the Gibbs energy of transfer from the aqueous lye to the polymer

$$\Delta_{P}^{W}\phi_{K^{+}}^{o}=\frac{\Delta G_{\mathrm{tr},K^{+}}^{o,w\to p}}{z_{K^{+}}F}$$ (26)

The Gibbs energy of transfer of K^+^ and OH^−^ cannot be found in the literature, but these values have been listed for a series of water | organic solvents, and to give an order of magnitude, the standard transfer potentials of K^+^ and OH^−^ are about 0.5 and −0.5 V, respectively, for the water | 1,2-dichloroethane interface [31], meaning that even if the Gibbs energy of transfer of the salt is about 100 kJ·mol^−1^, the distribution potential is negligeable, and we can consider that the lye | ISM is not polarized. The de-hydration of KOH in the organic polymer may, of course, not be complete. Indeed, it has been shown computationally that mono- and di-hydrate complexes of the potassium cation in nonpolar media are rather stable species [32].

**Fig. 5.**
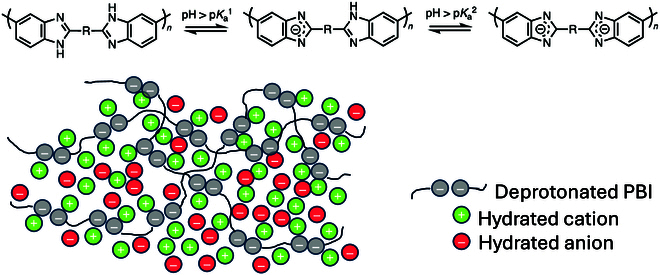
Schematic illustration of a PBI membrane at high pH in the presence of hydrated KOH.

Alternatively, the ISM can be considered as a cation-exchange membrane. Indeed, in the case of PBI, strong bases like KOH can deprotonate the benzimidazole amine groups (p*K*_a_ = 12.8), leading to the formation of the corresponding benzimidazolides anions [33]. As for zirconia particles illustrated in Fig. 2, the negative charges on the polymer backbone will be compensated by a Helmholtz layer of potassium ions, thereby transforming the polymer as a weak anion exchanger. This is schematized in Fig. 5.

The role of cations is indeed interesting and a strategy to make the PBI membrane a K^+^ conductor is to incorporate metal ions such as Zn^2+^, as proposed for assembling zinc–iron flow batteries [34].

**Fig. 6.**
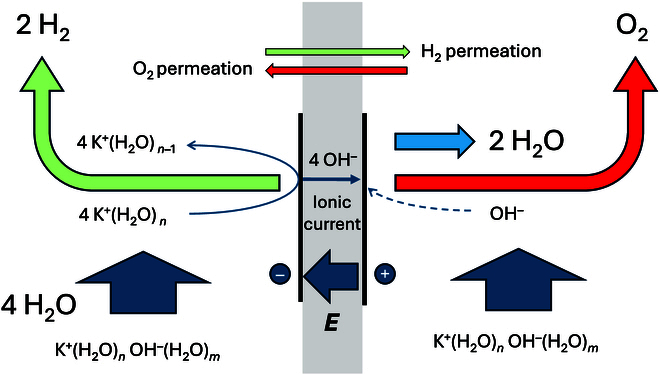
Fluxes across an ISM during alkaline water electrolysis.

Figure 6 summarizes the different fluxes during electrolysis using a water-solvating membrane. The major differences with Fig. 3 stem from a very reduced flux of salt and water across the membrane, and a reduced gas permeation.

## AEM-Based Systems

### Membrane electrode assemblies

Ion-exchange membranes being either cationic or anionic play an important role for the design of compact electrolyzers. Proton conducting membranes were developed initially for hydrogen fuel cells where the protons produced by hydrogen oxidation carry the current to the cathode where oxygen is being reduced. With time, they were used to assemble PEM (polymer exchange membrane) electrolyzers. The advantages of PEM electrolyzers include the compactness, i.e., a low footprint, the only reactant is pure water, and they can be operated at relatively high pressure, e.g., 30 bars. The disadvantages stem mainly from the materials used such as precious metal electrocatalysts, e.g., iridium oxide for the anode and fluorinated proton conducting membranes, which can potentially generate polyfluoroalkyl substances (PFAS). The electrode connectors are mainly composed of metallic or carbon foam electrodes [also porous transport layers (PTLs) or gas diffusion layers (GDLs)]. The membrane is sandwiched between 2 electrodes, forming a membrane electrode assembly (MEA) onto which electrocatalysts are deposited. Microporous layers are sometimes used between the PTL and the MEA.

Another class of ion-exchange membrane-based systems is the AEM electrolyzers. These systems are gaining a lot of interest as they share some of the advantages of the PEM electrolyzers in terms of compactness and ease of use. They also rely on transition metal electrocatalysts and on more readily available anion-exchange polymers. The major limitation of AEM electrolyzers is the polymer membrane chemical stability with a lack of data on their lifetime, and therefore, the calculations of the operating costs remain elusive.

The major difference between an alkaline electrolyzer and an AEM electrolyzer is the concentration of KOH used: 7 M in the former case and generally 1 M in the latter case. The KOH solutions are therefore much less corrosive and more user-friendly.

Nonetheless, as recently reviewed by Mamlouk [35], AEM can degrade due to both mechanical and chemical stress. The former stems from the membrane swelling and the applied pressure on the membrane such that a tensile strength of >15 MPa is usually recommended to avoid the formation of cracks and possible intrusion of fibers from the porous transport layer (PTL). The latter stems mainly from the nucleophilic attack of OH^−^, not so much on the polymer backbone but on the cationic group by substitution (SN_2_) or elimination (E_2_) reactions. The presence of radicals during electrolysis is also a cause of membrane degradation, which leads to the loss of cationic headgroups. Recently, membranes featuring γ-amine-piperidinium-functionalized polystyrene, featuring a carbazole segment to tune ion-exchange capacities, have shown robustness features to envisage industrial applications [36].

When using AEM, the transport number of hydroxide ideally tends toward unity. The conductivity target expressed by the US Department of Energy is more than 100 mS·cm^−1^.

### Hydroxide transport in AEM

As discussed above, studying ion motion in confined solutions such as AEM membranes remains a challenge as water dynamics and ion motion occur over time and length scales ranging from femtosecond to picosecond for the former and microseconds to milliseconds for the latter. Recently, 2D IR spectroscopy and semiclassical simulations were used to examine how water molecules are arranged into successive solvation shells and the interplay with bromide ion transport [37].

Chen et al. [38] have proposed a model for hydroxide solvation and transport in AEMs. They distinguished the so-called vehicular mechanism associated to classical ionic motion in solution (Brownian motion) and the Grotthuss mechanism (structural transport) involving bond breaking and bond making, establishing that the former contributes considerably more than the latter for hydroxide transport in the AEM. This is different to PEM systems, which show the opposite for proton transport. They also noted that the activation energy barrier for hydroxide diffusion in the AEM is greater than that for proton diffusion in PEMs, implying a more significant enhancement of ion transport in the AEM at elevated temperatures.

In 2018, using computer simulations, Dong et al. [39] have shown that the Grotthuss mechanism is more important in very confined environment and bottlenecks in the polymer structure. More recently, Dubey and Daschakraborty [40] have further observed a significant role of hydration level and temperature in the mechanism of vehicular diffusion. In the presence of water and at a higher temperature, both the hydroxide ion and water diffuse via mainly small steps, while at a lower hydration level and temperature, the diffusion becomes more governed by the jump-diffusion mechanism involving large steps.

A key parameter in the design of an AEM is the degree of amination and water uptake that promotes the transport of water and hydroxide anions in interconnected water channels, but at the detriment of the mechanical stability of the membrane. In the case of AEMs, the concentration of fixed cationic groups is given in meq·g^−1^ usually in the range 1 to 3 meq^−1^ [41]. This corresponds to a concentration of less than 3 M (mol·L_membrane_^−1^) and a water fraction of less than 0.5. Although the water content is higher than for an ISM, the presence of fixed cationic sites favors hydroxide transport by jump diffusion. In AEM, it is very likely that all types of hydroxide transport occur concomitantly: viscous Brownian motion with ion–ion interactions, Grotthuss-type mechanisms involving both free water molecules or electrolyte chains, and jump diffusion between fixed sites.

Recently, Tomasino et al. [42] have computed that a degree of 30% amination was optimum. Numerical simulations have also been used to characterize commercial membranes such as Sustanion (Dioxide materials), which is an imidazolium-based membrane, for which the authors concluded that vehicular diffusion is the predominant mechanism for chloride transport by diffusion, but contributes only 15% for hydroxide diffusivity [43]. Poly(arylene indole piperidinium) AEMs have also been studied theoretically, in particular the influence of the side chains. The results clearly show that flexible side chains favor OH^−^ transport due to the overlapping of the hydration shells, and how the molecular structure of the polymer influences the formation of water clusters [44]. Very recently, Menga et al. [45] have shown by simulations that the addition of crown-ether moieties in poly(arylene piperidinium) improves the OH^−^ mobility by increasing the solvation shell of the cationic groups facilitating the formation of continuous and uniform ion transport channels.

If numerical simulations provide insightful information at the molecular level, the gap with industrial applications of AEM water electrolysis remains important, as in the “real world” the large current densities used generate large electric fields and high fluxes of ions and water molecules. The presence of nanobubbles further complicates the membrane transport processes.

### The role of the salts in AEM

**Fig. 7.**
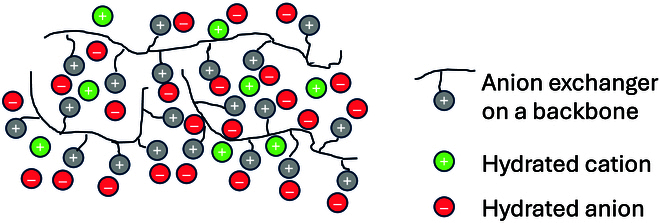
Schematic illustration of an ion-exchange polymer in the presence of hydrated KOH. Whereas a PBI ISM carries a negative charge on its backbone that must be compensated by K^+^ to allow OH^−^ transport, AEM membranes carry positive charges either on its backbone or on pendant groups.

Park et al. [46] recently studied a series of alkali metal hydroxides. The transport number of the alkali cations was measured, and the results obtained showed that naturally the transport numbers of the cations increase with the electrolyte concentration, the membrane being not ideally selective, but also varied with the nature of the cations: *t*_LI+_ = 0.131, *t*_Na+_ = 0.145, *t*_K+_ = 0.160, *t*_Cs+_ = 0.174 for a 0.01 M solution, and *t*_LI+_ = 0.201, *t*_Na+_ = 0.214, *t*_K+_ = 0.221, *t*_Cs+_ = 0.230 for a 0.1 M solution. It can be observed that the less hydrated the cation, the higher the transport number, in line with the predictions of Eqs. 11 and 12 for a simple binary electrolyte. They also showed in a previous publication that the addition of non-electroactive nitrate salts to an AEM electrolyzer operating with a 0.01 M NaOH solution altered the water electrolysis performance [47]. They emphasized the role of cations in delivering water to a dry cathode and concluded that alkaline water electrolysis with low pH (e.g., pH 12) but with an additional high ionic strength electrolyte may be an interesting strategy for long-term electrolysis durability at low electrolysis overpotential (1.73 V for 800 h), since excessive hydroxide concentrations can be avoided. Figure 7 schematically illustrates an AEM and the presence of mobile hydroxide ions.

### The role of the ionomers

When assembling PEM or AEM electrolyzers, ionomers are used at the membrane | electrode interface. The role of ionomers in ion-exchange membrane electrolyzer designs is often discussed in relation to the improvement of the catalyst layer structure and the increased adhesion between the MEA and the porous electrode support, hence a reduced contact resistance and finally to improve the stability of the catalysts by providing a stable environment. Ionomers can play a role both in the anodic compartment and on the cathodic side. For the anodic side, the key role of the ionomer is to provide a right balance between ionic and electronic conductivity between the catalysts and the electrode feeder. For example, it was recently shown that a variation of 25% in electrolyzer performance can be obtained just by varying the ionomer content [48]. On the cathodic side, it is important to distinguish electrolyzers operating a dry cathode mode and those where the cathodic compartment is fed with the electrolyte solution. In the former case, the ionomers play a key role as a “sponge” to keep a certain water content and maintain ionic conductivity. Counterintuitively, Titheridge et al. [49] have shown that the cation exchanger Nafion can play a useful role as a cathodic ionomer, consistently performing better than other ionomers tested in systems circulating KOH. This was attributed to Nafion providing more free volume for the electrolyte. This topic raises still many questions, as a recent study [50] has clearly shown that for supporting electrolyte-fed AEM devices, the ionomer is not really useful for ion conduction through the catalyst layer as the ionic strength is large enough to ensure the current. However, the ionomer was shown to play a key role for the catalyst layer structure and its stability. Intermediate ionomer contents lead to the lowest overpotentials, highest effective surface areas, and lowest catalyst layer resistances. The stability was linked to catalyst adhesion and ionomer loss. One open question is to know if the ionomer solution should be similar to the precursors used to synthetize the ion-exchange membrane. This study suggests that different chemistries can be used, but it remains difficult to say if it is a general conclusion. It is interesting to notice that ionomers on both side of the membrane allow the electrolysis of pure water [51].

Water management in AEM was studied by in situ neutron imaging, showing that a higher ion-exchange capacity of the ionomer provided a better water retention, reducing the drying out of the cathode at high current densities [52]. Indeed, dry cathode operation leads to a water distribution characterized with a high-water content at the anode and therefore a gradient inside the MEA toward the dry cathode. Further, this gradient increases with current density, as water is consumed in the hydrogen evolution reaction at the cathode and is further transported to the anode by both electro-osmotic flow and dragging by the migrating hydroxide anions.

**Fig. 8.**
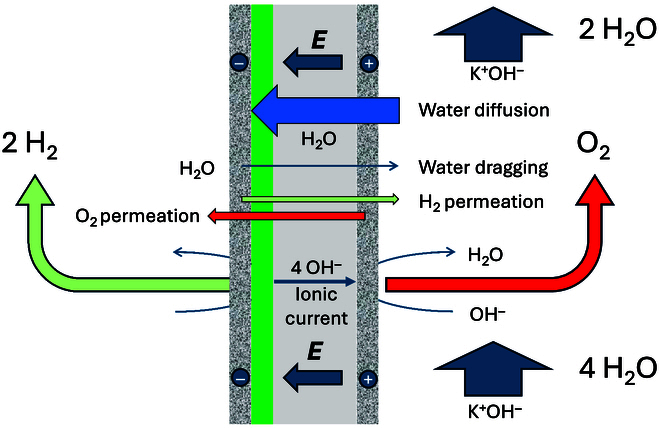
Fluxes across an AEM during alkaline water electrolysis. The cathode compartment can be exclusively dry or wet with water or lye circulation. The green layer represents the ionomer layer on the porous cathode.

Figure 8 shows a schematic view of a porous cathode AEM cell comprising porous electrodes, metallic or carbon based, sandwiching the membrane. The major difference here is the importance of the water fluxes to the cathode where it is reduced to produce H_2_ and OH^−^. In AEM electrolysis, K^+^ plays no active role, but its solvation helps to increase the water content.

## Conclusion

To summarize from an ionic transport aspect, we can distinguish 3 main classes of membranes in water electrolysis under alkaline conditions.

First, the classical porous membrane where the solution inside membrane is similar in composition to that of the adjacent solutions, e.g., KOH (7 M). The ionic conductivity is then also very similar to that in the solutions, based on Brownian motion, with the major difference being the transport numbers, with that of the hydroxide anions increasing from 0.73 to unity. The presence of narrow pores (e.g., 150nm) and charged interfaces is likely to increase the proportion of hydroxide transport by a Grotthuss-type mechanism, i.e., hydrogen bonds making and breaking even in the absence of free water molecules. The high resistance observed experimentally stems mainly from ion–ion interactions acting as a friction force of the hydroxide anion mobility. The presence of negatively charged inorganic particles such as zirconia makes the membrane more hydrophilic, compensating the hydrophobic character of the polymer backbone, and favors swelling. As discussed above, the presence of a Helmholtz layer of quasi-immobile cations transforms the membrane from a weak cation exchanger to a weak anion exchanger.

The second class of membranes includes so-called ISMs. The ionic composition inside the membranes may differ from that of the adjacent solutions. The concentration of KOH inside the membrane is likely less than in solution due to the hydrophobicity of the polymer, but this would need to be verified experimentally. The low dielectric constant of the membrane should enhance ion–ion interactions and favor the motion of hydroxide by a Grotthuss-type mechanism even in the absence of free water molecules. In the case of PBI, the polymer backbone becomes negatively charged. Again, the presence of a Helmholtz layer of cations transforms the membrane from a weak cation exchanger to a weak anion exchanger and makes the membrane more hydrophilic.

To account for the fast transport of OH^−^ in porous and ion-solvating membranes, a Grotthuss-type mechanism involving the hydrated potassium cations may be worth speculating. In a classical Grotthuss mechanism, a chain of free water molecules is needed for the proton or hydroxide to transfer from one end of the chain to the other, just by a series of bond breaking and bond formation. In highly concentrated solutions being more like a “molten salt of hydrated ions”, can we consider a Grotthuss-type mechanism involving the hydration shell of the cations? This could occur either by strengthening the hydrogen bond network or by involving the formation of ion pairs K^+^OH^−^. Indeed, it has been reported using polarization-resolved femtosecond-IR spectroscopy for NaOD/D_2_O solutions that at high concentrations, OD^−^ turns the solution in a “semi-rigid hydrogen-bond network” [53].

All in all, the quest for high current density electrolyzers will require low resistance membranes like ion solvating or anion exchange where fast hydroxide motions are not limited by ion–ion interactions. Of course, at the end of the day, only membranes that will be able to offer low electrical resistances and efficient gas barriers while answering to optimal economic criteria, i.e., cost and robustness, will be able to emerge at the industrial scale.
