# Supplementary material for: Ionic Transport Aspects of Water Electrolysis in Alkaline Media
Source: Research (Wash D C). 2025 Aug 28;8:0788. doi: 10.34133/research.0788 (PMC12393796; doi:10.34133/research.0788)
Supplement: Supplementary 1 — Supplementary Text Figs. S1 to S3 [file research.0788.f1.docx]

SUPPLEMENTARY MATERIALS


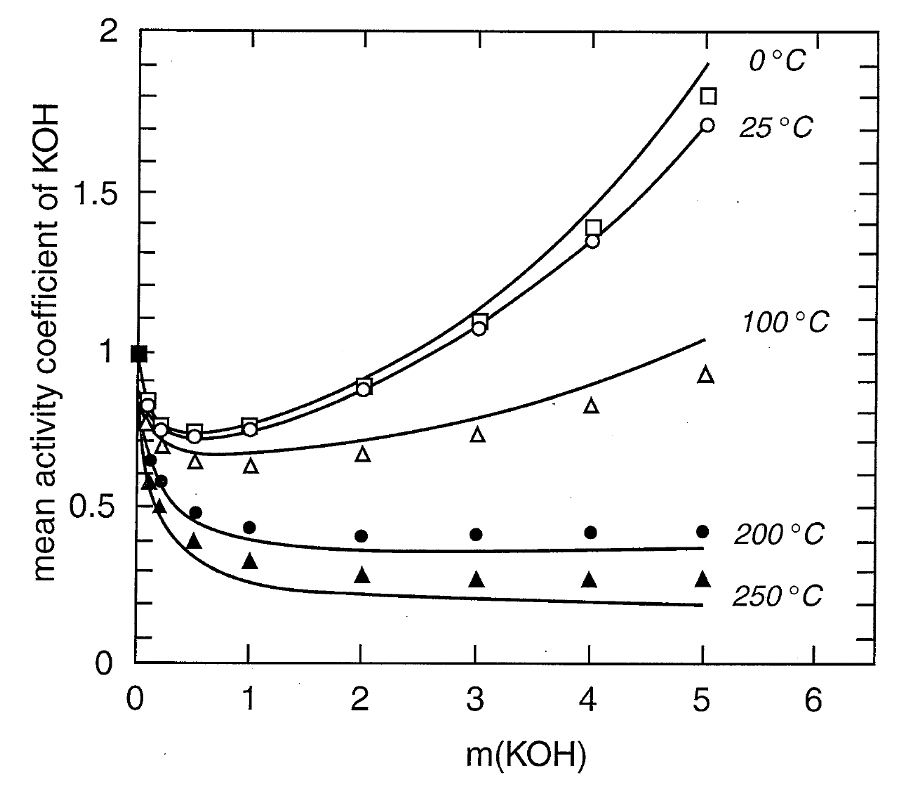


***Fig S1.*** Mean activity coefficient of KOH in water as a function of molality from ref. [S1]


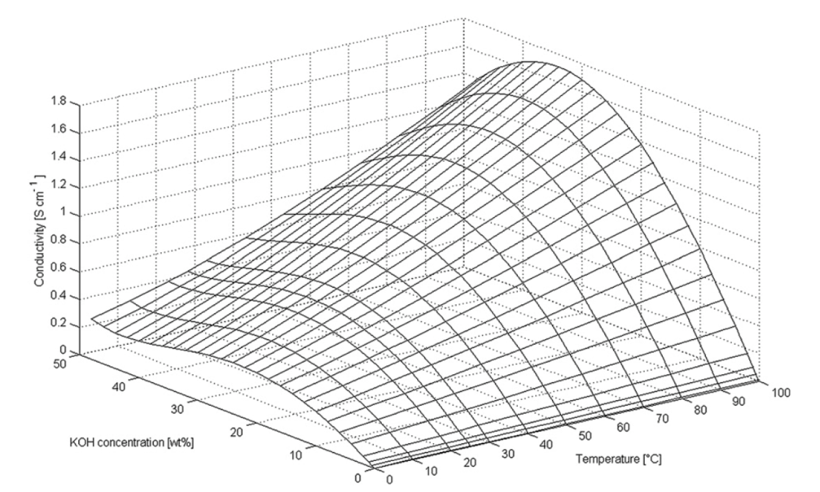


***Fig. S2:*** Conductivity of KOH solutions from ref. [S2].


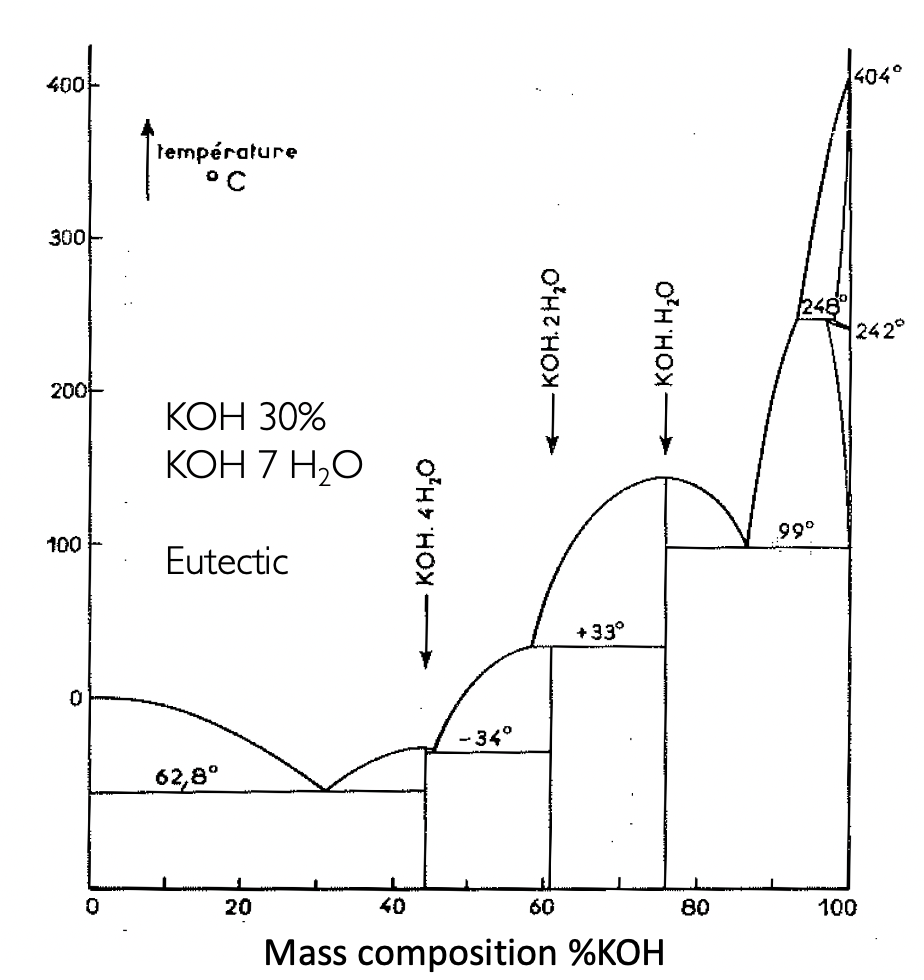


***Fig. S3:*** Phase diagram for water-KOH adapted from ref. [S3]

**Stefan-Maxwell equations:**

*Friction coefficients:* At steady state, the friction forces by the species *j* on a moving species *i* are equal to the driving force generated by a gradient of electrochemical potential


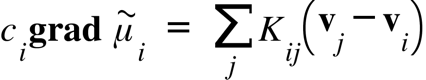
 (S1)

where *c_i_* is the concentration of the species *i*,
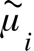
 the electrochemical potential and **v** the velocity vector. *K_ij_* is the friction coefficient of *j* on *i*. Following Onsager reciprocity law, we have *K_ij._* = *K_ji_* . As discussed by Bennion *et al.*^8^, the different friction coefficients can be obtained by carrying out independent experiments using for example pressurised cells and conductivity measurements. The friction of the solvent on the ions is by nature due to ion-dipole interactions and the friction between the different ions is mainly due to coulombic interaction.

The electrochemical potential, on the molar concentration scale, is given by:


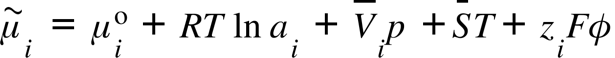
 (S2)

where
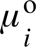
 is the standard chemical potential, *a* the activity,
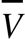
 and
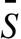
 are the partial molar volume and partial molar entropy, respectively. *F* is the Faraday constant and *φ* the Galvani potential, also called inner potential of the phase.

We shall discuss first here the classical example of a binary electrolyte like KOH in water transporting the current through a porous membrane. In this case, we can write eq.(1) for the ions K^+^ & OH^–^ and the water molecules


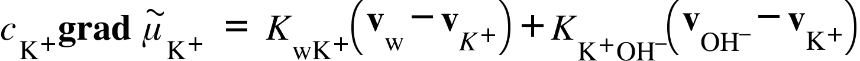
 (S3)


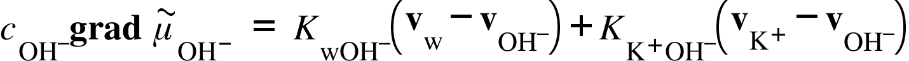

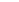
 (S4)


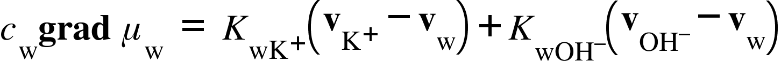
 (S5)

The ion-ion friction coefficient,
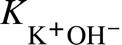
, is concentration dependant increasing with the concentration. Indeed, as predicted by Kohlrausch law the conductivity decreases with the concentration.

*Dilute solutions:* In dilute solutions, the ion-ion interactions can be neglected and the term
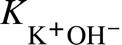
becomes nil. Furthermore, if the solutions are static (**v**_w_ = 0), we have from the definition of the flux of the species *i*


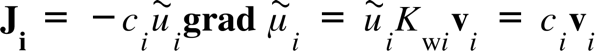
 (S6)

where
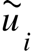
 is the electrochemical mobility, which is always positive. It is related to the diffusion coefficient by the Einstein relation


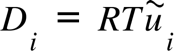
 (S7)

Hence, in dilute solutions, the friction coefficients with the solvent are simply defined as


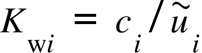
. (S8)

Also, the ionic conductivity in dilute solutions is directly proportional to the electrochemical mobility


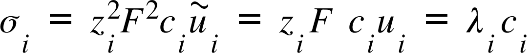
 (S9)

where *u_i_* is the electrophoretic mobility
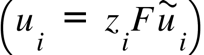
.

*General case:* In a first approximation, we shall assume electroneutrality in the solution within the membrane, hence equal concentration of cations and anions, defined as the concentration of KOH. The current density, **j** , through the membrane results from the opposite fluxes of the cations and anions in the membrane.


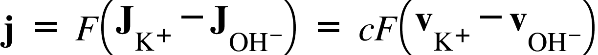
 (S10)

where *c* is the concentration of KOH, *F* Faraday’s constant and **J** the respective ionic fluxes. The flux of water through the membrane is


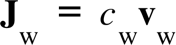
 (S11)

where *c*_w_ is the free water concentration. This term is usually taken as constant in dilute solutions but may vary here in highly concentrated alkaline solutions where the concentration of free water molecules is small. By re-arrangement of eq.(3-4), the ionic fluxes can be expressed as a function of the current density, **j ,** and of the water transport.


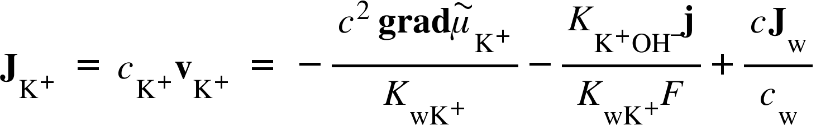
 (S12)


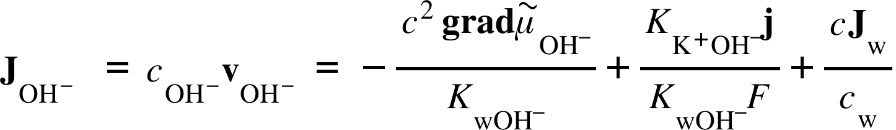
 (S13)

Prior to electrolysis when a lye of identical composition is circulated on either side of the diaphragm, the different fluxes are nil. As soon as the electrical current is applied, the different gradients will be established and potassium transfer needs also to be considered, unless
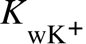
 is very large. Using eq. (12 & 13), the current density can be expressed as a function of the driving forces for the ions


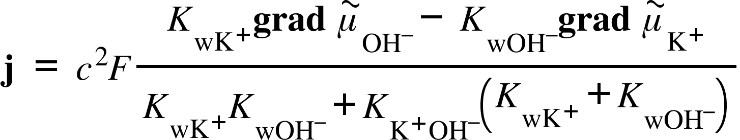
 (S14)

The influence of water transport from the analyte to the catholyte appears only on the friction coefficients of the solvent on the ions
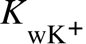
 and
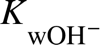
.

Considering that ion motion is only due to diffusion-migration, neglecting the osmotic and thermal effects, the driving forces for the ions are due to a gradient of concentrations and a gradient of Galvani potential *φ*.


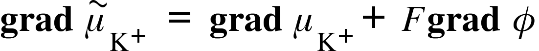
 (S15)


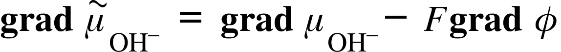
 (S16)

The conductivity, *σ* , inside the membrane is classically defined from the current density and the electric field, **E** , acting on the membrane.


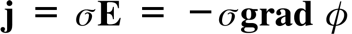
 (S17)

In a homogeneous porous diaphragm, the electric field can be considered in a first approximation as constant and the Galvani potential varies linearly across the membrane. From eq. (14-16), the conductivity can be calculated as a function of the different friction coefficients.


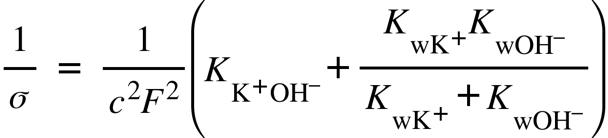
 (S18)

As we have seen above, in dilute solutions
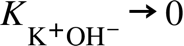
 , and eq. (18) reads simply


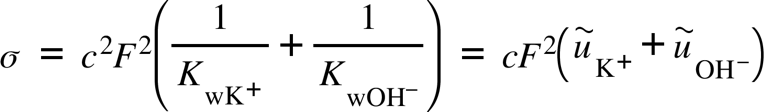
 (S19)

We recover the classical definition of electrolyte conductivity for dilute solutions.

If we further consider that the cation-water friction is greater than that of anion-water
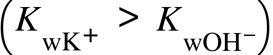
, for example considering a Grotthuss mechanism for OH^–^, eq. (18) reduces to


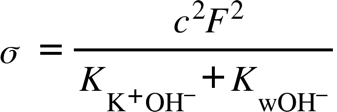
 (S20)

Using the conductivity data of figure S1, we can see that the conductivity increases with concentration but not in a quadratic manner as both the ion-ion friction and the solvent-OH^–^ friction also increase. When the conductivity decreases with concentration, the dominant term in eq. (20) is
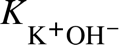
 simply due to the lack of free water molecules.

*Transport numbers:* The transport numbers of the ions are defined^6^ such that


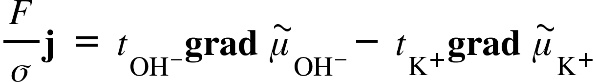
 (S21)

They represent the fraction of the current carried by the two ions. From eq.(14), we obtain the simply expression


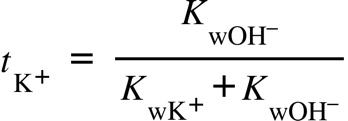
 (S22)


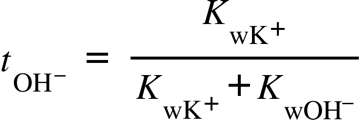
 (S23)

As discussed, hydroxide anions are more mobile than the potassium cations
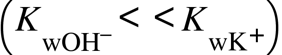
 and then.
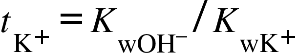
 is small and
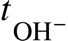
 tends to unity.

*KOH diffusion:* If we want to consider the diffusion of KOH from the catholyte to the anolyte, we can use the definition of the chemical potential of KOH,


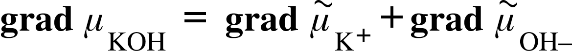
 (S24)

Starting form eq. (21) with
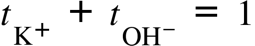
 , we can write


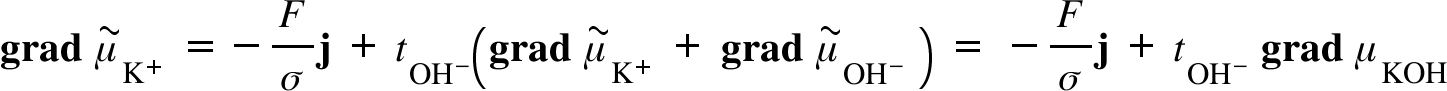


(S25)

Similarly


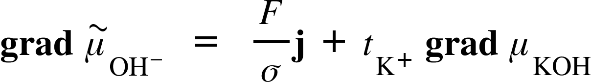
 (S26)

Using eq. (12 & 13), we obtain


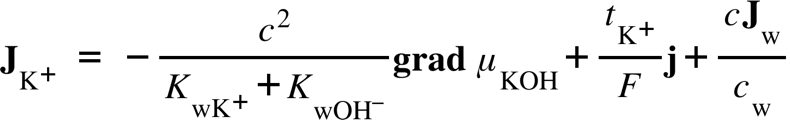
 (S27)


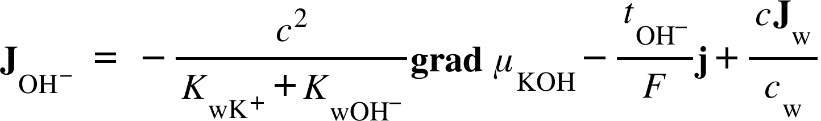
 (S28)

These equations show that the flux of potassium is mainly a flux of diffusion, and a flux associated to the transport of water. The latter can be a flux of diffusion from anolyte to catholyte or a convective flux associated to pressure difference. Conversely, the flux of hydroxide is mainly a migration flux associated to the passage of the current.

We can define the flux of KOH as


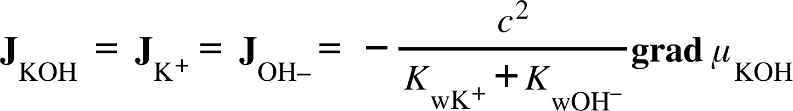
 (S29)

It is interesting to notice in figure S1 that at 80°C the activity coefficient of KOH is rather constant and close to unity.

**SI References:**

S1. Christov C, Moller M, Chemical equilibrium model of solution behavior and solubility in the H-Na-K-OH-Cl-HSO_4_-SO_4_-H_2_O system to high concentration and temperature, Geochim. Cosmochim. Act., 2004; 68: 1309–1331, 2004

S2. Allebrod F, Chatzichristodoulou C, Mollerup PL and Mogensen MB, Electrical conductivity measurements of aqueous and immobilized potassium hydroxide, Int. J. Hydrog. Energy , 2012; 37: 16505 -16514

S3. Pascal P, Nouveau traité de chimie minérale, Tome II, , Masson 1963
